# Cerebellar cortical neuron responses evoked from the spinal border cell tract

**DOI:** 10.3389/fncir.2013.00157

**Published:** 2013-10-08

**Authors:** Pontus Geborek, Anton Spanne, Fredrik Bengtsson, Henrik Jörntell

**Affiliations:** Neural Basis of Sensorimotor Control, Department of Experimental Medical Science, Lund UniversityLund, Sweden

**Keywords:** spinocerebellar tracts, granule cells, mossy fibers, Purkinje cells, golgi cells, interneurons, spinal cord, cerebellar cortex

## Abstract

Spinocerebellar systems are likely to be crucial for cerebellar hallmark functions such as coordination. However, in terms of cerebellar functional analyses, these are perhaps among the least explored systems. The aim of the present study is to achieve activation of a single component of the spinocerebellar systems and to explore to what extent it can influence the spike output of granule cells, Golgi cells, molecular layer (ML) interneurons (stellate and basket cells) and Purkinje cells (PCs). For this purpose, we took advantage of a unique arrangement discovered in neuroanatomical studies, in which the spinal border cell (SBC) component of the ventral spinocerebellar system was found to be the only spinocerebellar tract which ascends in the contralateral lateral funiculus (coLF) and have terminations in sublobulus C1 of the paramedian lobule in the posterior cerebellum. Using electrical stimulation of this tract, we find a subset of the cerebellar cortical neurons in this region to be moderately or powerfully activated. For example, some of our granule cells displayed high intensity responses whereas the majority of the granule cells displayed no response at all. The finding that more than half of the PCs were activated by stimulation of the SBC tract indicated that this system is capable of directly influencing cerebellar cortical output. The implications of these findings for the view of the integrative functions of the cerebellar cortex are discussed.

## Introduction

The spinocerebellar tracts constitute a major part of the total mossy fiber input to the cerebellum (Oscarsson, 1973) and are likely to be crucial components in the cerebellar function of coordination (Spanne and Jorntell, 2013). However, there is a multitude of different spinocerebellar pathways (Oscarsson, 1973; Matsushita et al., 1979; Matsushita and Ikeda, 1980) and there is today limited knowledge of the potency with which individual pathways can affect the different neurons of the cerebellar circuitry.

The purpose of the present study is to characterize the responses of cerebellar cortical neurons to mossy fiber input from the spinal border cell (SBC) tract. The SBC tract is one of the spinocerebellar tracts, specifically one of the subcomponents of the ventral spinocerebellar tract (Matsushita et al., 1979; Matsushita and Ikeda, 1980). In the posterior lobe of the cerebellum, SBC terminations are believed to be concentrated to, or even limited to, the sublobulus C1 of the paramedian lobule (Matsushita and Ikeda, 1980; Matsushita and Yaginuma, 1989). Since this region does not appear to receive input from other components of the ventral spinocerebellar tract (Matsushita and Ikeda, 1980), the SBC tract should be the only spinocerebellar tract which ascends in the contralateral lateral funiculus (coLF) and have terminations in sublobulus C1. Hence, stimulation of the coLF and recording cerebellar neuron responses would pose a unique opportunity to record the effects of one spinocerebellar tract in isolation. This can substantially facilitate the interpretation of how the responses are generated, as opposed to most *in vivo* studies of cerebellar cortex where multiple parallel pathways with widely different conduction times and synaptic linkages are activated.

In the present study, we use mid-thoracic electrical stimulation of the coLF, verified to antidromically activate SBCs, and record the responses of the cerebellar cortical neurons in sublobulus C1. We find only a small fraction of the granule cells to be activated by coLF stimulation, but many of these granule cells have strong spike responses. Among Golgi cells, molecular layer (ML) interneurons and Purkinje cells (PCs), somewhat less than half of the neurons display weak to moderate spike responses. We conclude that a single spinocerebellar tract appears to be capable of driving cerebellar cortex activity and hence influence the cortical output to the deep cerebellar nucleus.

## Materials and methods

All experiments (*N* = 20) were made in the acute decerebrated preparation of the cat. The cats were prepared as previously described (Ekerot and Jorntell, 2001; Jorntell and Ekerot, 2002, 2003). Briefly, following an initial anesthesia with propofol (Diprivan^®^ Zeneca Ltd, Macclesfield Cheshire, UK), the animals were decerebrated at the intercollicular level and the anesthesia was discontinued. The animals were artificially ventilated and the end-expiratory CO_2_, blood pressure and rectal temperature were continuously monitored and maintained within physiological limits. Mounting in a stereotaxic frame, drainage of cerebrospinal fluid, pneumothorax and clamping the spinal processes of a few cervical and lumbar vertebral bodies served to increase the mechanical stability of the preparation. To verify that the animal was decerebrated, we made EEG recordings using a silver ball electrode placed on the surface of the superior parietal cortex. Our EEG recordings were characterized by a background of periodic 1–4 Hz oscillatory activity, periodically interrupted by large-amplitude 7–14 Hz spindle oscillations lasting for 0.5 sec or more. These forms of EEG activities are normally associated with deep stages of sleep (Niedermayer and Lopes da Silva, 1993). The pattern of EEG activity and the blood pressure remained stable, also on noxious stimulation, throughout experiments (see also, Jorntell and Ekerot, 2006).

### Recordings and stimulation

Before recordings, the bone and dura covering the posterior part of the left cerebellar paramedian lobule was removed. Laminectomies were made at the level of spinal segments T7–T9 and at the level of the spinal segments L3–L5. We aimed to make *in vivo* patch clamp recordings from neurons of the cerebellar cortex, in the sublobulus C1. This was done with patch clamp pipettes pulled to 6–19 MOhm potassium-gluconate based internal solution. Obtaining whole cell recordings from granule cells in the sublobule C1, however, proved more difficult than in the more accessible anterior lobe (Jorntell and Ekerot, 2006). Therefore the present paper includes only loose cell-attached recordings. The general procedures for patch pipette recordings in the granule layer in this preparation have been described previously (Jorntell and Ekerot, 2006). The seal resistances of the recordings of the present material were between 200–2000 MOhm. A HEKA EPC 800 patch clamp amplifier, set to current clamp, was used to amplify the responses from the micropipettes. The signal was converted to a digital signal using the analog-to-digital converter Power 1401 mkII from Cambridge Electronic Design (CED, Cambridge, UK). Extracellular metal electrode recordings (tungsten-in-glass microelectrodes with conical metal tips of 10–30 μm exposed length, with a tip diameter of well below 1 μm) were made from the granule cell layer (GCL) in the sublobulus C1 and SBCs at the L4 segment of the spinal cord, respectively. In order to be able to analyze the activity with a computer the analog-to-digital converter (Power 1401 mkII) from CED was used. The neural responses were sampled at 100 KHz and recorded continuously with the software Spike 2 from CED. The signal from the amplifiers was split between the Power 1401 mkII, and a NAD 302 stereo amplifier. The NAD 302 was used to listen to the analogue signal for monitoring activity during the experiment. The lateral funiculus, the cerebellar cortex and the SBC region were stimulated with tungsten microelectrodes with exposed tips of 30–120 μm. The Digitimer DS3 (Isolated constant current stimulator/stimulus isolator, Digitimer Ltd, Letchworth Garden City, UK) were used in order to deliver reproducible square stimulus pulses with a constant current. The Power 1401 mkII was used as an event timer for the Digitimer stimulators.

### Data processing

All neural data was converted from analogue to digital form using the Power 1401 mkII from CED. The software Spike 2 from CED was used to record the digital data. Spike 2 was also used to sort spike activity from noise. Spike shapes were required to have a characteristic spike shape as well as a signal to noise ratio of at least 1:3 in order to verify that it was a neural response rather than ambient noise being analyzed. Peristimulus histograms were made using Matlab. The local field potential analysis was done using a kernel estimation method to interpolate between the recording points in the cerebellum sublobulus C1.

### Cortical cell recordings within the medial part of sublobulus C1

We made loose cell-attached extracellular recordings from granule cells and Golgi cells within the GCL of the medial sublobulus C1, and from PCs and ML interneurons in the overlying Purkinje cell layer (PCL) and ML. The definition of a unit as a granule cell was primarily done using the characteristic spike signature of granule cells, in particular the presence of interspike intervals of < 2.0 ms (Van Dijck et al., 2013), and by verifying that they were located in the granule layer based on field potential recordings (Bengtsson and Jorntell, 2007) and by keeping track of the depths at which PCs were encountered in each experiment for each plane of penetration (this could be done since each experiment involved a high number of electrode tracks). In some experiments, recorded granule cells were recovered morphologically and verified to have the characteristic morphology of granule cells (*N* = 4). In these experiments, the recording solution of the patch pipettes contained neurobiotin (1.8%) to obtain juxtacellular labeling (Pinault, 1996) of the granule cells we recorded from. In order to increase probability of staining electroporation was done at the end of recording, after all other electrophysiological tests were done. Electroporation was made with 0.1–0.4 nA square pulses with a 300 ms duty, and a 200 ms rest phase, repeated for at least 1 min. At the end of these experiments, the animals were sacrificed by injecting a lethal dose of 3 ml barbiturate and subsequently perfused with paraformaldehyde (4%). The posterior lobe of the cerebellum was excised and stored in paraformaldehyde for up to a week, before the cerebella was sectioned into 60 μm sagittal slices. The slices were incubated with streptavidin conjugated with Alexa 488 Fluor (Molecular Probes, Invitrogen Inc.) and mounted for visualization under the confocal microscope (Zeiss 310 LSM and Zeiss 510 LSM).

### Quantification of the responses in cerebellar cortical neurons

In order to quantify the responses evoked by coLF stimulation we made peristimulus histograms of the evoked spike responses with 1 ms bin widths. For each histogram, an increase of the response by more than 1 S.D. from the 100 ms prestimulus baseline activity for at least three out of five consecutive bins was taken as an evoked response. Assuming that the frequencies of the Peristimulus time histogram (PSTH) bins are normally distributed, the likelihood of reaching above one standard deviation in a single bin would be approximately 15.9%. By choosing three out of the five PSTH bins as the limit for detection, the risk of a false positive can be calculated to be 3.07%. This is the combined probability for all permutations where at least three out of five bins have values higher than one standard deviation above the mean value. This test was a good way to measure both slow broad based responses as well as rapid sharp responses. Only responses at 10 ms or shorter response latency time from effective stimulation were considered, since responses evoked at longer latency times were considered unlikely to be due to activation of the SBC tract. In order to calculate the mean net change in firing frequency, spontaneous activity, if it existed, was subtracted from the evoked response and the mean change in spike firing frequency over five consecutive bins was calculated. For the systematic data quantification, we included only the responses evoked by the three pulse stimulations (3 pulses at 3 ms interval) of the coLF.

The experimental procedures were approved in advance by the local Swedish Animal Research Ethics Committee.

## Results

**Figure 1:**
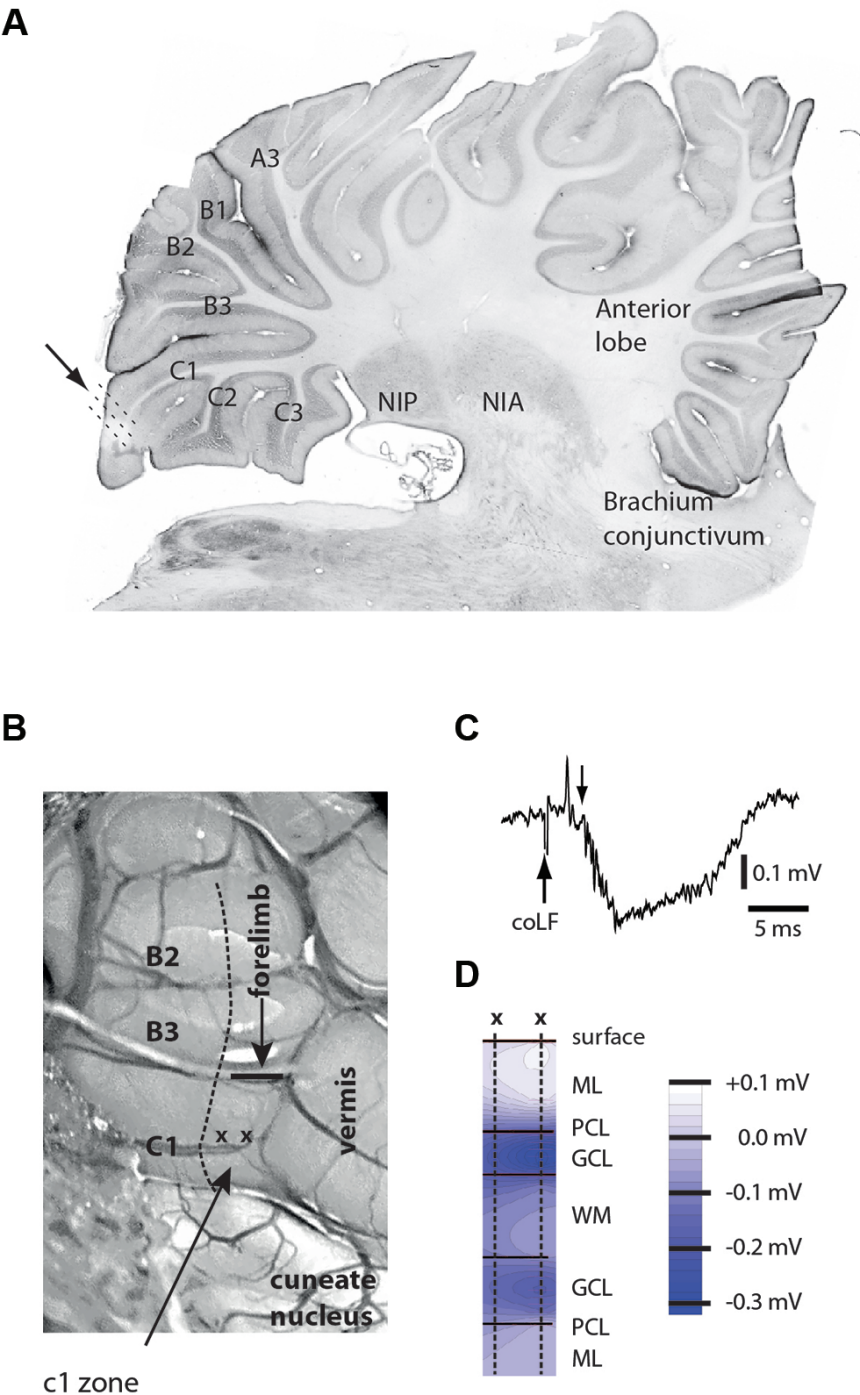
**Anatomical and physiological identification of the recording region.** **(A)** Microelectrode and patch clamp recording electrodes were inserted at an angle of 30–45˚ relative to the surface of sublobulus C1 (arrow and dotted lines). Letters and numerals refer to the nomenclature of the sublobules in the paramedian lobule (Matsushita and Ikeda, 1980). NIP, nucleus interpositus posterior; NIA, nucleus interpositus anterior. **(B)** Surface view of the posterior part of the paramedian lobule, viewed at an angle of approximately 30˚. Sublobules are indicated to the left. The arrow points to the C1 zone (not to be confused with the sublobule C1), within which all unit recordings were made. Also indicated are the location of the vermis and the caudal termination (horizontal bar) of the forelimb representation of the C1 zone. The horizontal bar is also a calibration for a 1 mm distance. **(C)** Sample field potential response, evoked in the GCL by coLF stimulation at 300 μA. Downward arrow indicates the onset of the evoked field potential. The preceding volley reflects evoked ascending activity from the brain stem. **(D)** Depth profiles of responses evoked in medial sublobulus C1 by single pulse stimulation of coLF at 0.5 mA. ML, PCL, GCL, WM.

We aimed to make loose-patch recordings from the cerebellar cortical neurons of sublobule C1 (Figure 1A), since patch clamp recordings are essentially required to obtain granule cell recordings *in vivo* (Jorntell and Ekerot, 2006). However, before recording sessions commenced, we delineated the functional organization of the climbing fiber inputs of the paramedian lobule, within which the sublobule C1 is located. We found that the more rostral parts of the C1 zone (not to be confused with the sublobule C1) in the paramedian lobule received climbing fiber input from the ipsilateral distal forelimb, as previously described (Armstrong et al., 1971a,b; Trott and Apps, 1993), and the caudal termination of this representation was found to be a reliable indicator of where the anatomically defined sublobule C1 and the representation of the coLF input began (Figure 1B). This part of the cerebellum was sometimes also found to receive a weak climbing fiber input from the distal ipsilateral hindlimb.

### Field potential recordings of coLF responses in sublobulus C1

In order to further maximize our chances of finding cortical neurons activated by putative SBC input, we first conducted a field potential study of responses evoked by stimulation of the coFL. Focussing on sublobule C1, we recorded the distribution of evoked field potentials (Figures 1C,D). The distribution of maximal mossy fiber field potentials was investigated in eight experiments. In all cases, we found that maximal field potentials evoked by coLF stimulation were located in the most medial part of the sublobule C1. The response latency times of the onset of these potentials were 4.3 +/−0.1 ms (mean +/− standard deviation) for field potentials evoked from Th8–Th9 (*N* = 8).

### Antidromic activation of SBCs from the cerebellar cortex and the coLF

**Figure 2:**
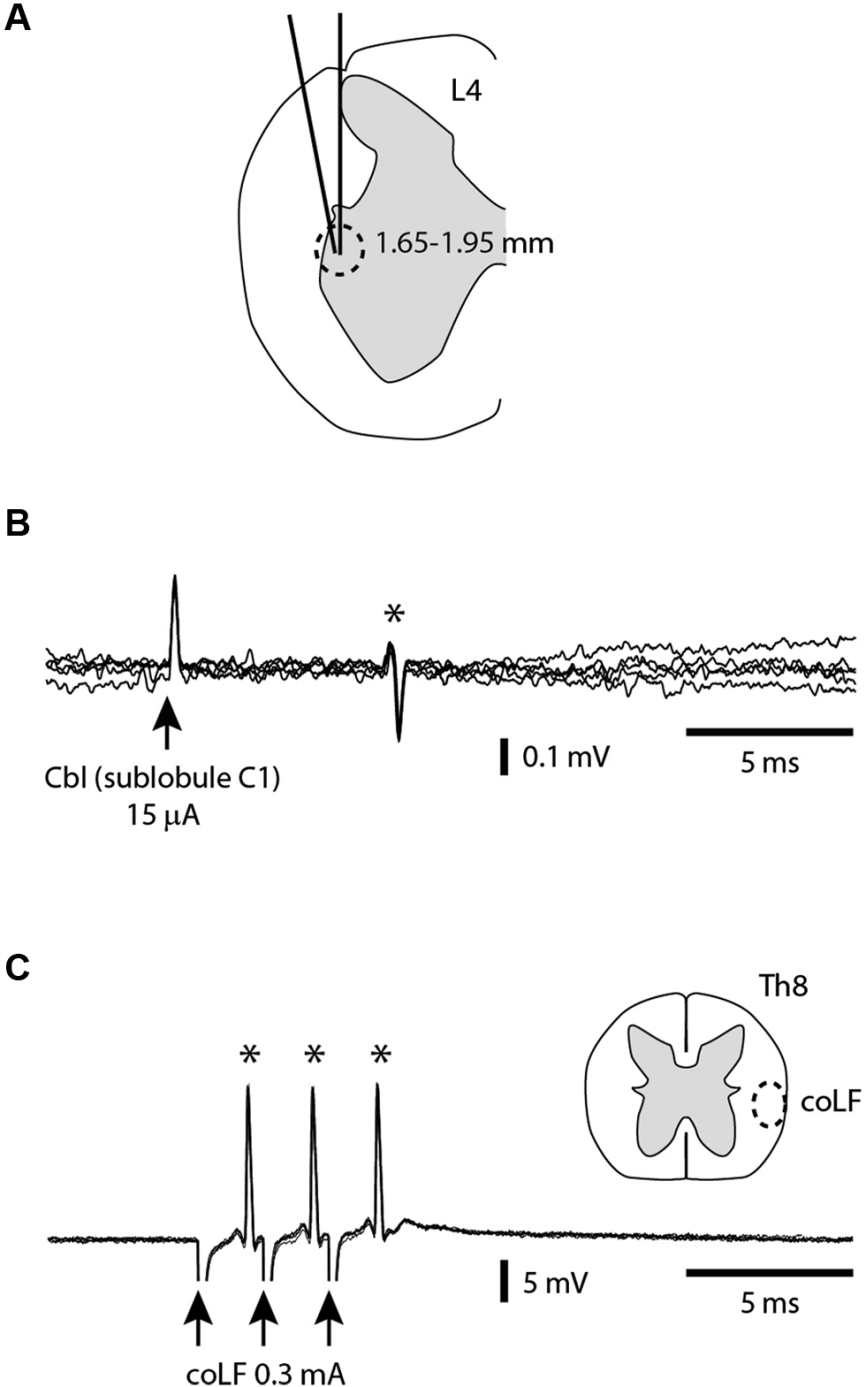
**Antidromic activation of SBCs from the sublobule C1 and the coLF.** **(A)** A cross-section of the L4 segment (from Shrestha et al., 2012a) indicating the two alternative orientation of the electrode tracks and the depths at which the SBCs were found. **(B)** Example of SBC extracellular unit (*) antidromically activated from sublobule C1 in the cerebellum. **(C)** Example of SBC unit (*) antidromically activated from the coLF. Also indicated is the targeted location in the Th8 segment of the coLF stimulation electrode, based on Matsushita et al. (1979); Matsushita and Ikeda (1980).

In order to verify that our coLF stimulation was effective in activating the SBCs, we made direct tungsten electrode recordings from SBCs (*N* = 25) in the L4 segment of the spinal cord and tested whether they were antidromically activated. To obtain SBC recordings, we used two alternate types of tracks (Figure 2A). In one of the approaches, the electrode had 0˚ angle in the mediolateral plane and entered the spinal cord at the same mediolateral level as the dorsal root entrance. In the second approach, the electrode was tilted 10˚ in the mediolateral plane and entered the spinal cord a few 100 μms lateral to the dorsal root entrance. With both approaches, the electrode travelled through white matter (WM), where we monitored the ambient noise level by sound, the impalement of axons by the sharp tip of the electrode, and the lack of somatic neuronal unit recordings, all the way down to a depth of 1.5–1.7 mm, where noise indicative of neuronal units started to appear. Since our recordings were obtained from the first neurons encountered as we entered grey matter substance in the dorsal part of the ventral horn, they would by definition correspond to SBCs (Matsushita and Ikeda, 1980; Shrestha et al., 2012b). All SBC units encountered were located at a depth of 1.65–1.95 mm. Their antidromic activation was verified by recording a constant response latency time of the SBC spike at both single and triple pulse stimulation (three pulses at 3 ms interpulse interval) using interstimulus intervals of 300 ms (Figures 2A,B). Without antidromic stimulation, they were all (*N* = 25) completely silent.

For a subset of the recorded SBCs (*N* = 9), we investigated the points of minimal threshold for antidromic activation from the sublobuli B2, B3 and C1 (see Figure 1A) of the cerebellar cortex. This was done by using a tungsten stimulation electrode. As we tracked for low threshold points, the stimulation electrode was switched to recording mode so that the type of cerebellar layer the electrode was located in could be monitored. For each SBC, we managed to find points of minimal thresholds for antidromic activation within the cerebellar cortex (Figure 2B) with minimal effective intensities below 30 μA, in a few cases below 10 μA. These low threshold points were always located within the GCL of the medial part of sublobulus C1, confirming the findings that we previously made with field potential recordings that the SBC tract terminated primarily medially within the sublobule C1 (Figure 1). The response latency times for antidromic activation of SBCs from the cerebellar cortex was 6.0 +/−1.9 ms, with a range of 4.2–9.8 ms (*N* = 9).

The next step was to verify that the SBCs were also antidromically activated by the coLF stimulation (Figure 2C) that we used for evoking activity in the cerebellar cortical neurons. A separate tungsten stimulation electrode was lowered about 1.8–2.0 mm into the ventrolateral quadrant of the spinal cord at the thoracic level 8. The stimulation intensity was adjusted to find the threshold for antidromic activation, which could be as low as 30 μA. A fixed response latency time (with a standard deviation of 0.0 ms in all cases, *N* = 25) already at threshold stimulation was taken as an indication that these neurons were antidromically rather than synaptically activated. To increase the likelihood that these neurons were not synaptically activated by the stimulation, we compared the highest possible subthreshold stimulation intensity (with no response) with the threshold intensities. The differences were less than 5% in all cases. However, collision tests, where a spontaneous spike blocks the arrival of the antidromically activated spike, were not possible to perform since the SBC neurons in our preparation were completely silent and exhibited no spontaneous spiking. Nevertheless, we believe that the other measures we did obtain are sufficient to make it highly likely that the SBC neurons were indeed antidromically activated by the coLF stimulation, as previously shown (Jankowska et al., 2011a,b; Shrestha et al., 2012a,b). The highest thresholds for antidromic activation of SBCs using coLF stimulation was 0.3–0.5 mA, and this was also the intensity chosen for activation of cerebellar cortical neurons in the subsequent parts of the paper. For coLF stimulation, the response latency times for antidromic activation of SBCs was 1.9 +/−0.8 ms, with a range of 1.2–2.8 ms (*N* = 25).

### Responses of granule cells to coLF stimulation

**Figure 3:**
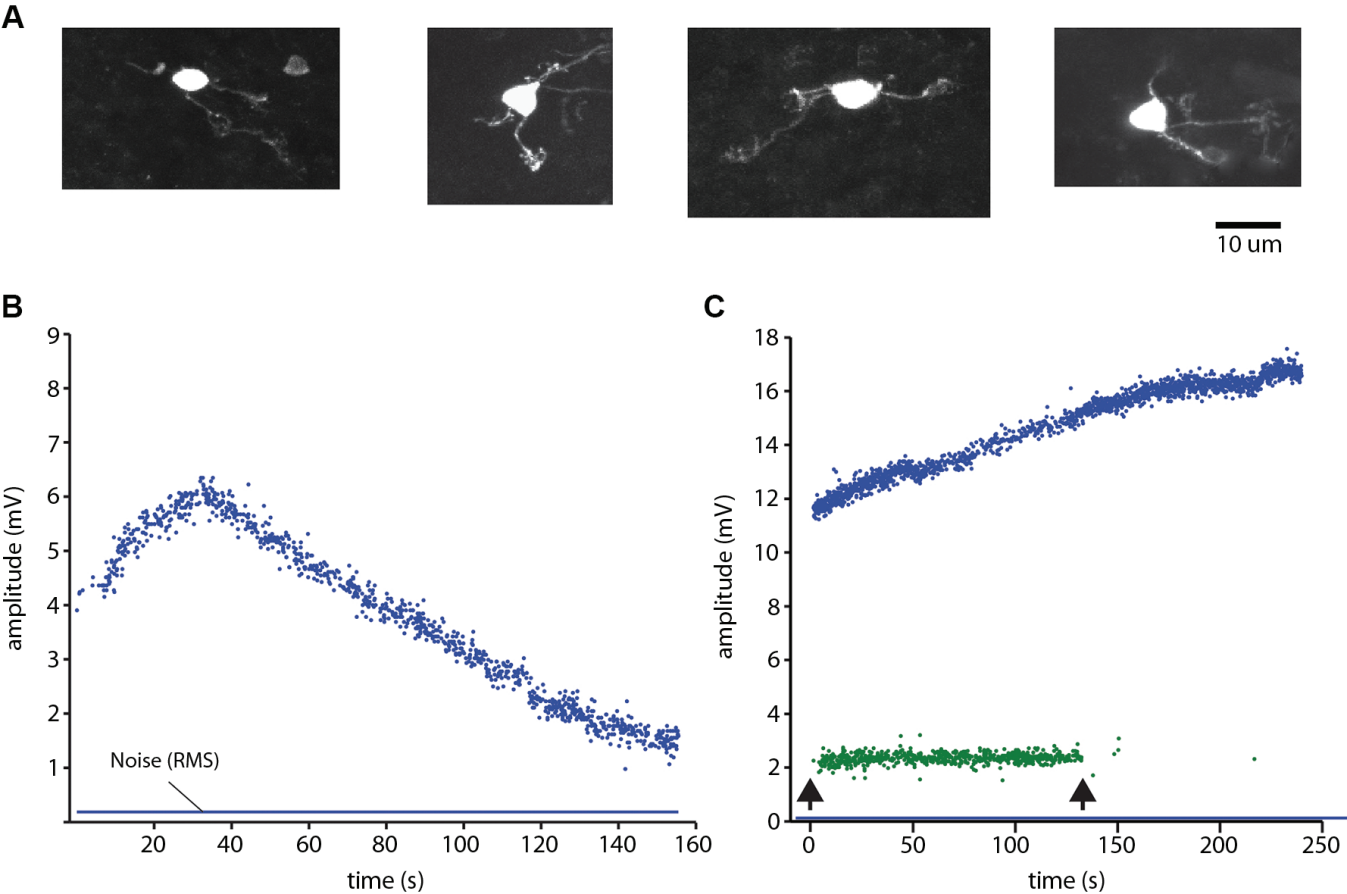
**Granule cell identification.** **(A)** Examples of morphologically identified granule cells recovered from the sublobule C1. **(B)** Plot of the peak to peak amplitude of a spike recording of a granule cell in cell-attached mode. Note the variation over time of the spike amplitude. Blue line indicates the root mean square of the noise level of the recordings. Spike activity was evoked by ongoing coLF stimulation. **(C)** Single electrode-parallel recording of a Golgi cell (blue) and a granule cell (green) (Bengtsson and Jorntell, 2009a; Bengtsson et al., 2013). Arrows indicate onset and offset, respectively, of the coLF stimulation.

Granule cells (Figure 3A) and the other neurons were recorded in the cell-attached mode. The identification of neurons as single units was made on basis of single spike shape (using templates in software) and a consistent spike amplitude. During the experiments we continuously monitored the spike signals on loudspeakers and computer screens to make sure that the isolated unit was a single unit. Against the background that the cell-attached mode has been reported to in rare cases generate dual cell recordings (Bengtsson and Jorntell, 2009a; Bengtsson et al., 2013), we here illustrate how we can be sure that we recorded from single granule cells. Figure 3B illustrates the amplitude of a granule cell spike, which could vary over time. Figure 3C illustrates a rare example of a dual cell recording in the GCL, from a Golgi cell and a granule cell. Here, too, the spike amplitude varied over time, but the variation was different for the two neurons, which was an additional means to verify that the two neurons were distinct units and that single neuron recordings were derived from a single neuron. Note that this measurement started already as we approached the neuron, before the seal was established but when we could detect spike activity, which was monitored throughout all electrode tracks. Hence, the possibility that any neural recording labeled as unitary was rather a dual recording must be minimal or negligible.

**Figure 4:**
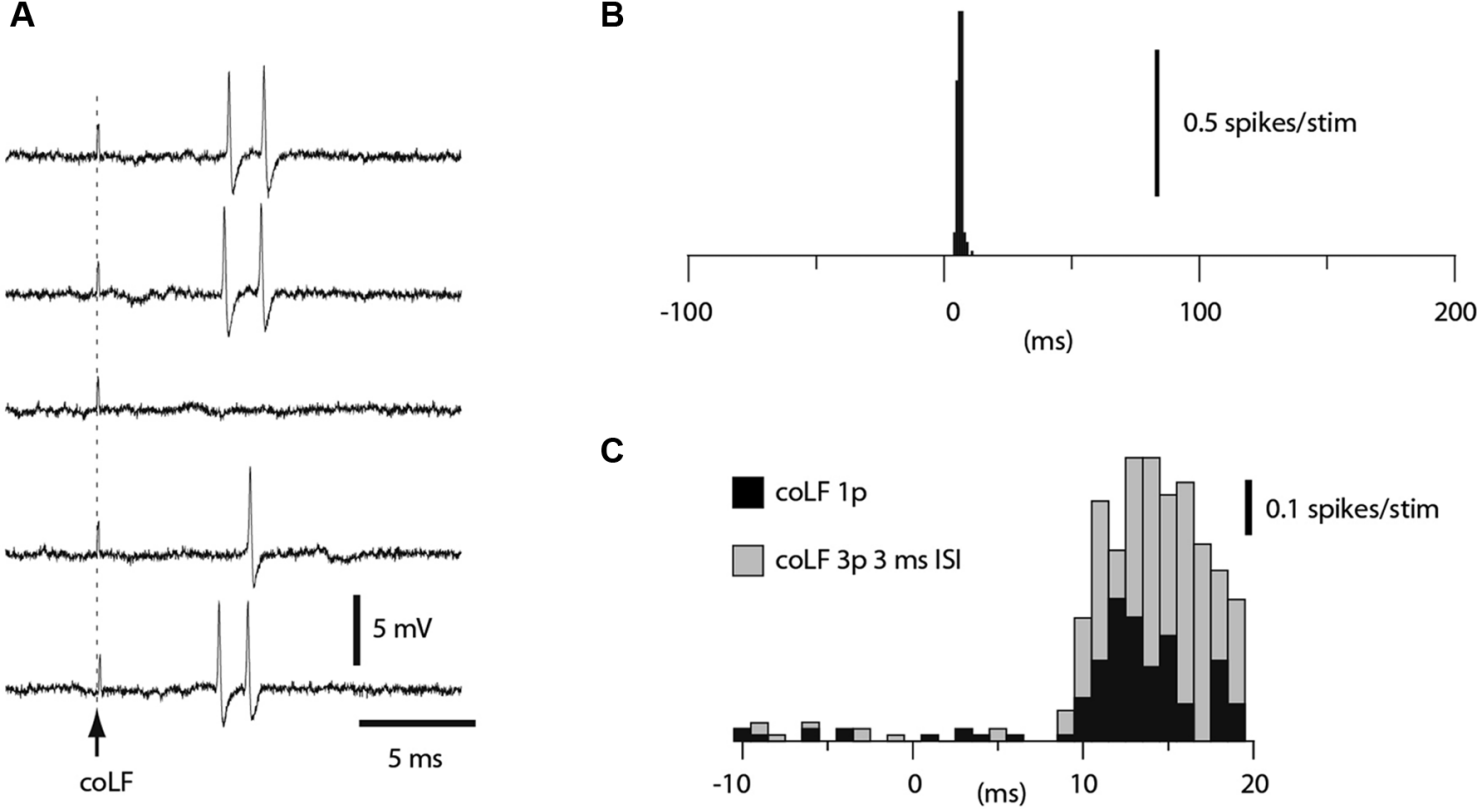
**Granule cell responses evoked by coLF stimulation.** **(A)** Loose cell-attached extracellular recording of a granule cell responding to coLF stimulation at 0.5 mA. **(B)** Peristimulus histogram for the granule cell in A. **(C)** Peristimulus histogram of the responses of another granule cell to coLF stimulation using single (black bars) and three pulse stimulation (3 ms interpulse interval) (grey bars), respectively. The bin width in the histograms was 1 ms.

A subset of the granule cells recorded (*N* = 164) responded to coLF stimulation with one or two spikes at a regular response latency time of 5–7 ms (Figure 4A). Such responses resulted in very sharp peaks in the peri-stimulus histograms (Figure 4B). In other granule cells, the spike responses were also powerful but consisted of more variable spike response times, which created more broad-based responses in the peristimulus histograms (Figure 4C, black bars). In such cases, the apparent response latency time was typically longer. However, we routinely checked the granule cell responses also to triple pulse stimulation (3 ms interpulse interval). For longer latency responses, the triple pulse stimulation typically provided additional responses at the same time or even before the response evoked by the single pulse stimulation, despite that the stimulations both started at time zero. Since the second pulse added to the response evoked by the single pulse (compare grey and black bars in Figure 4C), the response latency time from the second pulse was taken as the *effective response latency* in these cases. Based on the antidromic response latency times of the SBCs, which fell in the range of 4–10 ms (Figure 2), only granule cell responses that had an effective response latency time of less than 11 ms were considered to be due to activation of SBCs. In this group of granule cells, the effective response latency time was 6.5 +/−1.2 ms (range 4.5–9.0 ms, *N* = 29). The magnitude of the responses was measured from the first 5 ms of the response evoked by the 3 pulse stimulation. Based on the response magnitudes, we could segregate the granule cells into two groups (*p* < 0.05, *t*-test); those responding with an average spike intensity of 90 Hz or more and those responding at less than 60 Hz. For the high-intensity group, the average firing frequency of the evoked response was 257 +/−98 Hz (*N* = 16). For the low-intensity group, the firing frequency of the evoked response was 46 +/−21 Hz (*N* = 13).

### Responses of Golgi cells, Purkinje cells and molecular layer interneurons to coLF stimulation

**Figure 5:**
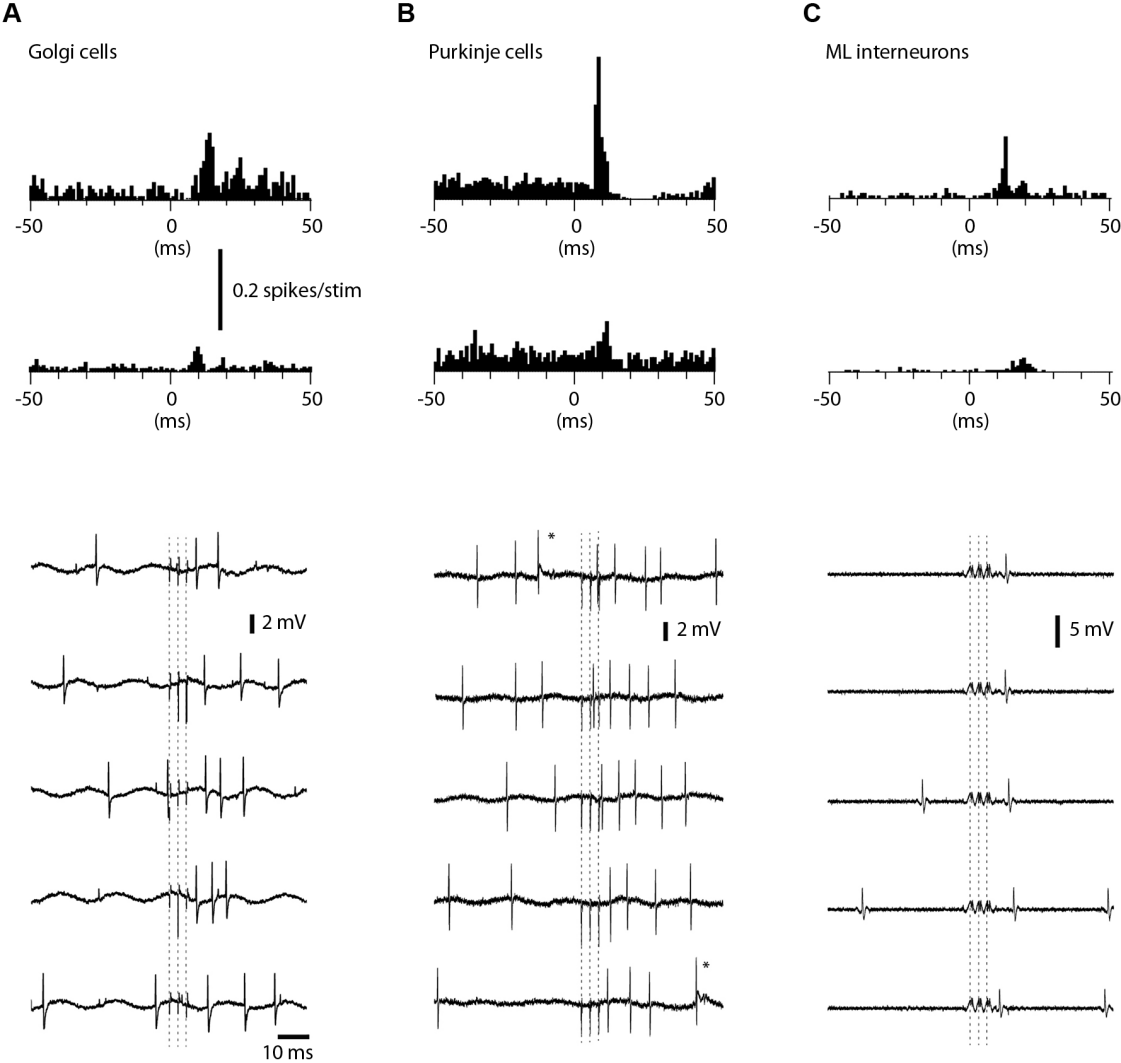
**Responses of Golgi cells, PCs and ML interneurons to coLF stimulation.** coLF triple pulse stimulation at 0.5 mA was used in all panels. At the top are peristimulus histograms with a bin width of 1 ms of the responses, at the bottom sample raw traces. **(A)** Peristimulus histograms for two examples of Golgi cells, one with a strong response and a second with a weaker response. Sample raw traces below are from the Golgi cell with strong responses. Dashed lines indicate time of stimulation (three pulses). **(B)** Peristimulus histograms for two examples of PCs (only simple spike activity was analyzed). Sample raw traces are from the PC with moderate response. Asterisks indicate complex spikes. **(C)** Peristimulus histograms for two examples of ML interneurons. Sample raw traces are from the ML with a strong response. Due to the very large artefacts from the coLF stimulation (due to the high seal in this recording) software filtering was applied in this case only.

We also recorded the evoked spike responses in Golgi cells (*N* = 27), PCs (*N* = 16) and ML interneurons (*N* = 11) in the medial part of sublobule C1. Golgi cells were identified by their location in the granule layer (see description for granule cells in Section Materials and Methods; briefly, the definition relies on the characteristic polarity changes of evoked mossy fiber field potential responses and the location of the PCL where the characteristic complex spikes can be recorded (Eccles et al., 1967; Bengtsson and Jorntell, 2007)) and their long tuning distances as well as their firing characteristics (Van Dijck et al., 2013), PCs were identified by the presence of complex spikes whereas interneurons were identified by their location in the ML interneurons and the absence of complex spikes (Jorntell and Ekerot, 2011). Complex spikes were separated from simple spikes by their distinct secondary depolarization and secondary spikelets within 1–5 ms after the initial spike (cf. Figure 5B).

Among Golgi cells, direct responses to coLF stimulation (Figure 5A) were more common (11/27) than among granule cells, possibly reflecting the large dendritic trees and more widespread input sampling of these cells. Similar to granule cells, the intensity of the response could vary substantially between Golgi cells (Figure 5A). The effective response latency times were 6.5 +/−1.6 ms (*N* = 11), also this value was similar to that of granule cells. The net evoked responses were 62 +/−27 Hz (*N* = 11), with a considerable range (4–82 Hz). Also some PCs in this region responded quite powerfully to coLF stimulation (Figure 5B), whereas other PCs had weaker input. Only 6 out of the 16 PCs recorded lacked detectable responses to coLF stimulation. The effective response latency time was 8.7 +/−1.6 ms and the net evoked responses were 40 +/−32 Hz (range 10–103 Hz). For ML interneurons (Figure 5C), 5 of the 11 recorded neurons had responses with an effective response latency time of 8.8 +/−1.6 ms and a net response of 42 +/−26 Hz (range 8–93 Hz).

## Discussion

The present paper is the first investigation of the responses evoked in all the major types of cerebellar cortical neurons from a putative single component of the spinocerebellar systems. The approach of directly activating mossy fibers greatly facilitates the interpretation of how the responses are generated as compared to other studies where more complex inputs (i.e., peripheral activation of multiple parallel pathways) or behaviorally generated spike discharges have been analyzed. The analysis of the responses of cerebellar cortical neurons to the direct activation of the SBC mossy fiber pathway indicated that these responses are relatively straight forward reflections of the input, although the synaptic weights of the input may vary across the population. Some of the neurons responded with very powerful responses and the responses evoked in the PCs suggest that the information conveyed by a single component of the spinocerebellar pathways can readily make its way to the cerebellar cortical output. The implications of our findings in relation to our understanding of cerebellar function in general, and the Marr-Albus family of ideas of cerebellar granule layer function in particular, are discussed.

### Transmission of the spinocerebellar mossy fiber information through the cerebellar cortex

Different neurons had different effective response latency times, which is compatible with our observations that the antidromic response latency times of the SBCs varied considerably, similar to the antidromic response latency times for other components of the ventral spinocerebellar tract (Geborek et al., 2013). Quite possibly, some granule cells received SBC inputs from units with similar conduction velocity, which would be expected to result in very sharp response profiles in the histograms as in Figure 4B, whereas others received inputs from less well synchronized SBC inputs whereby the response profiles in the histograms would become broader (Figure 4C). Since granule cells typically have a relatively wide gap between the resting membrane potential and firing threshold and requires the summation of three or four mossy fiber synapses to fire (Chadderton et al., 2004; Jorntell and Ekerot, 2006; Duguid et al., 2012), even a low-amplitude broad based response could be considered a strong response. Notably, since we used direct electrical stimulation of a population of mossy fibers, the input generated is artificially synchronous and does of course not reflect the spatiotemporal patterns of activity that these mossy fibers would display during behavior. Nevertheless, it does provide a measure of how effective the synaptic input is and whether it can be transmitted to downstream neurons. The range of different intensities in the responses suggests that we recorded from Golgi cells, PCs and ML interneurons with different locations relative to the clusters of granule cells receiving strong activation from the coLF stimulation. The fact that the granule cell responses were transmitted, suggests that this population of granule cells (i.e., those activated by the SBC tract) provide relatively effective synapses to the other cortical neuron types.

### Implications for models of cerebellar cortical function

For granule cells, the average response frequency of 260 Hz (in the group of granule cells labeled high-intensity responders) is a very strong response, although higher intensity responses have been reported using more natural types of activation of the mossy fiber input (Jorntell and Ekerot, 2006). Nevertheless, for a stimulation that would be expected to set up only 3 spikes at 333 Hz in each of the activated mossy fibers, such response intensities in granule cells is compatible with that most or all of the mossy fiber inputs to these granule cells were activated from the stimulated tract, in agreement with our previous analysis of granule cells in the cerebellar C3 zone of the anterior lobe (Jorntell and Ekerot, 2006; Bengtsson and Jorntell, 2009b). The present study could however not provide any direct evidence for this since we failed to obtain any intracellular recordings from the high-intensity responders. At any rate, the demonstration that the information is transmitted so powerfully through some of the granule cells, verified by the fact that we also recorded responses in the PCs and ML interneurons (Figure 4) has some important implications for the original Marr-Albus ideas of cerebellar granule layer processing (Marr, 1969; Albus, 1971). In Marr’s important theoretical paper, it was assumed that each of the four mossy fiber synapses that the granule cell received were needed to be activated in conjunction in order to trigger the output of the granule cells, which is also in agreement with findings from *in vitro* and *in vivo* studies (D’Angelo et al., 1995; Jorntell and Ekerot, 2006; Bengtsson and Jorntell, 2009b). However, Marr postulated that each of these mossy fibers carried unique information and it was only when input from these separate sources of information were driven coincidentally that the granule cell could be made to fire. Granule cell firing was hence believed to be a rare event and the idea of sparse coding in the GCL, which is a widespread notion in cerebellar theories, was a natural consequence of this line of reasoning. This part of the original Marr-Albus ideas would hence predict that a single mossy fiber pathway should not be capable of activating a granule cell. However, the present study tested this prediction and the result was that a single pathway is capable of activating a substantial part of the granule cells in the region. Some of them were in fact quite powerfully activated, and the output from these granule cells was also sufficient to activate many downstream neurons including nearly half of the PCs, which mediate the output of the cerebellar cortex. Similar conclusions have previously been drawn for a completely different system of the cerebellar cortex, i.e., the responses generated by the cuneate nucleus in the granule cells and the cortical neurons of the the C3 zone of the cerebellar anterior lobe (Dean et al., 2010). In the C3 zone, activation from single, small receptive fields from a single submodality evokes very intense granule cell responses, and the cells are easily sustaining firing frequencies of 100’s of Hz for many seconds if the peripheral input is delivered appropriately (Jorntell and Ekerot, 2006; Bengtsson and Jorntell, 2009b). The PCs of the C3 zone are powerfully driven by this input (Jorntell and Ekerot, 2002, 2011).

Nevertheless, in the present study, we also found a group of granule cells with weaker responses to SBC tract stimulation. For this group of cells, it cannot be excluded that some of the mossy fiber synapses that converged onto them were not activated by the SBC tract but derived from another, unidentified, input source. This would imply that the principle of similar coding convergence between mossy fibers and granule cells (Bengtsson and Jorntell, 2009b) does not always apply for 100% of the mossy fiber inputs to all granule cells but that there could be examples of granule cells that sample inputs from pathways that are not functionally identical. This principle has recently been demonstrated using cell-type specific projection mapping of external cuneate nucleus (ECN) and basilar pontine nucleus (BPN) mossy fibers to the cerebellar cortex of the mouse (Huang et al., 2013). ECN and BPN target mostly non-overlapping areas of the cerebellar cortex, but there are fringe zones in their terminations in which they overlap. In these fringe zones, Huang et al. demonstrated the existence of granule cells, which receive input from both BPN and ECN. An uncertainty in that paper is whether all the projections labeled were derived solely from ECN or BPN, since at least the termination of ECN mossy fibers were wider and less focused than those obtained with axon labeling of single, verified ECN cells (Quy et al., 2011). But the essence of their conclusion, that there exist granule cells which sample mossy fiber input from non-identical sources, seems undisputable. This would hence be compatible with our findings for the smaller group of granule cells that were low-intensity responders. It is important to recall that the existence of such granule cells does not change the conclusions with respect to the original Marr-Albus idea and cerebellar function discussed above—the fact that SBC tract stimulation alone can powerfully drive cerebellar output is sufficient to say that these original ideas cannot be correct in this respect.

### Alternative sources of mossy fiber inputs to sublobule C1

The region we recorded from could be defined as the physiological C1 zone of the paramedian lobule based on the climbing fiber responses that we recorded. The physiological C1 zone has a second representation in the cerebellar anterior lobe (Apps and Garwicz, 2005). Since the mossy fiber input to different parts of a single climbing fiber zone is at least partly branches from the same systems (Pijpers et al., 2006), the C1 zone of the sublobule C1 may be expected to receive mossy fiber inputs from similar systems as in the C1 zone of the anterior lobe. These systems are the bilateral ventral flexor reflex tract (bVFRT)-component of the lateral reticular nucleus (LRN; Clendenin et al., 1974; Ekerot, 1990), corticopontine input and dorsal column nucleus input from the trunk or hindlimb (Cooke et al., 1971; Gerrits et al., 1985). In addition, the neurons of the Clarke’s column of the thoracic segments, i.e., the thoracic component of the dorsal spinocerebellar tract (DSCT), has been shown to terminate in the sublobule C1 (Matsushita and Ikeda, 1980). None of these systems would be directly excited by coLF stimulation, as their fibers are all located on the ipsilateral side of the spinal cord: DSCT ascends ipsilaterally, the dorsal funiculus input ascends ipsilaterally, the bVFRT-input to the LRN ascends ipsilaterally, and the corticospinal fibers that activate the pontine nuclei is located in the ipsilateral dorsolateral funiculus, although a contribution from the uncrossed corticospinal fibers cannot be excluded. In the latter case, however, the fibers are few, are located ventrally in the spinal cord (Armand and Kuypers, 1980), and the input would involve an extra synaptic relay in the pons, which would imply later responses than the ones we observed. Taken together, this is an unlikely alternative. Activation of other descending motor command systems, such as the tecto- and vestibulospinal tracts, which could be located in the vicinity of the coLF, have bilateral terminations and therefore could activate one of the spinocerebellar systems targeting the sublobule C1, remains a likely alternative. However, this would involve extra synaptic delays and their responses would therefore be expected to occur later than inputs from the SBCs—in fact, in quite a few cases we saw substantial activations well beyond the 12 ms response latency limit we applied, which could correspond to this alternative route of cerebellar activation. For the granule cell spike responses we recorded, with an average effective latency time of 6 ms, direct activation of the ascending SBC axons remains the most probable route.

### Information conveyed by spinocerebellar tracts

Spinocerebellar neurons integrate information from descending motor command systems with sensory feedback information mediated by spinal premotor interneurons (summarized in Oscarsson, 1973; Jankowska et al., 2010; Hammar et al., 2011; Jankowska et al., 2011a,b; Krutki et al., 2011; Shrestha et al., 2012a,b; Spanne and Jorntell, 2013). Specifically, SBCs receive monosynaptic excitatory inputs from the reticulospinal tract, indirect information from rubro- and corticospinal tracts and primarily inhibitory inputs from interneurons activated by group I and group II muscle afferents (Hammar et al., 2011; Jankowska et al., 2011a; Shrestha et al., 2012a,b). The information conveyed seems to be sensory events and motor command components that applies to multiple limb segments and is therefore likely to be important for the function of coordination (Spanne and Jorntell, 2013). A possible explanation for the relatively powerful activation of the downstream cortical neurons from the SBC tract, despite that only a relatively small part of the granule cell population was activated by this input, is that this type of crucial signals for coordination are given high synaptic weights in a larger population of cortical neurons. In addition, the integration of sensory feedback with internal motor command signals makes these systems ideal substrates for the formation of internal models. The observation that PCs can signal in a fashion compatible with an internal model representation (Pasalar et al., 2006; Popa et al., 2012, 2013) could be due to the information conveyed by the spinocerebellar systems.

## Conflict of interest statement

The authors declare that the research was conducted in the absence of any commercial or financial relationships that could be construed as a potential conflict of interest.
